# Association between standing height and physical disability among U.S. adults aged 60 years and older: findings from NHANES 2015–2018

**DOI:** 10.1186/s12877-024-05100-3

**Published:** 2024-06-18

**Authors:** Shihong Wang, Zihua Yang, Xiwei Tan, Fengxia Lai, Ling Luo, Yuanlin Ding

**Affiliations:** 1 School of Public Health, Guangdong Medical University, Dongguan, Guangdong China; 2 School of Public Health and Emergency Management, South University of Science and Technology of China, Shenzhen, Guangdong China

**Keywords:** Physical disability, Standing height, Older adult, National Health and Nutrition Examination Survey

## Abstract

**Background:**

Physical disability is an important cause of affecting the quality of life in the elderly. The association between standing height and physical disability is less studied.

**Purpose:**

The purpose of this study is to investigate the possible link between standing height and physical disability among U.S. adults aged 60 years and older.

**Methods:**

The cross-sectional data were obtained from the US National Health and Nutrition Examination Survey (NHANES) 2015–2018. Physical disability was assessed by six questions: “Have serious difficulty hearing (SDH)?”, “Have serious difficulty seeing (SDS)?”, “Have serious difficulty concentrating (SDC)?”, “Have serious difficulty walking (SDW)?”, “Have difficulty dressing or bathing (DDB)?” and “Have difficulty doing errands alone (DDEA)?”. Responses to these questions were “yes” or “no”. Answer yes to one of the above six questions was identified as physical disability. Standing height (cm) was measured with an altimeter. Multivariate logistic regression was performed to examine the possible link between standing height and physical disability after adjustment for all covariates.

**Results:**

A total of 2624 participants aged ≥ 60 years were included in our study, including 1279 (48.7%) females and 1345 (51.3%) males. The mean age of participants was 69.41 ± 6.82 years. After adjusting for all potential confounders, the inverse relationship between standing height and all physical disability (APD) was statistically significant (*OR* = 0.976, 95*%CI*:0.957–0.995). In addition, among six types of physical disability (SDH, SDS, SDC, SDW, DDB, DDEA), standing height was also a protective factor for SDW (*OR* = 0.961, 95*%CI*:0.939–0.983) and DDEA (*OR* = 0.944, 95*%CI*:0.915–0.975) in the full-adjusted model.

**Conclusion:**

The cross-sectional population based study demonstrates that standing height is a protective factor for physical disability among U.S. adults aged 60 years and older.

## Introduction

Disability is defined as an umbrella term for impairments, activity limitations, and participation restrictions [1]. Physical disability is one of the most severe stages of disability, including loss of limbs, motor dysfunction, hearing impairment, visual impairment and etc [2]. People with physical disabilities are often at a disadvantage in their daily lives. The onset of physical disability is a dynamic process [3]. As health problems accumulate, people will eventually lose their mobility. Compared with the young, the elderly are more prone to physical disability due to the decline of physical function and immunity [4–6]. The World Health Organization (WHO) estimated the prevalence rate of disability was 10.2% in adults over 60 years around the world [5]. Meanwhile, one report said the prevalence rate of disability in mobility, hearing and vision in Americans aged 65 years and older was 26.9%, 14.9% and 6.6%, respectively [7]. Furthermore, physical disability is an important risk factor for many diseases in the elderly. Some previous studies have demonstrated strong associations between physical disability and the occurrence of depressive symptoms, diabetes, stroke and heart disease [8–11]. Physical disability has become a severe public health problem worldwide. Different factors may influence physical disability in different ages and in different populations. Therefore, identifying the risk factors related to physical disability in the elderly and implementing effective countermeasures are critical.

Standing height is one of the individual unmodifiable factor, so it is rarely the focus of the studies. Currently, standing height has been shown to have implications on human health and can be used to predict adverse outcomes [12]. For example, a previous cohort study showed standing height was inversely associated with gestational diabetes mellitus (GDM) [13]. For per 5-cm increase in standing height, the risk of GDM decreased by 19%. In addition, a meta-analysis including twelve cohort studies demonstrated greater height is linked with increased pancreatic cancer risk [14]. Each 5-cm height increment had a 7% increased risk of pancreatic cancer. Moreover, a review has also reported that height was an independent risk factor for atrial fibrillation [15]. However, to our knowledge, the association between standing height and physical disability in the elderly is unknown and has not been investigated. Unraveling the association will help to determine the prevention and treatment strategies of physical disability.

This study aimed to investigate the association between standing height and physical disability in the elderly. We explored the association by analyzing data from the US National Health and Nutrition Examination Survey (NHANES) 2015–2018.

## Materials and methods

### Study population

**Fig. 1:**
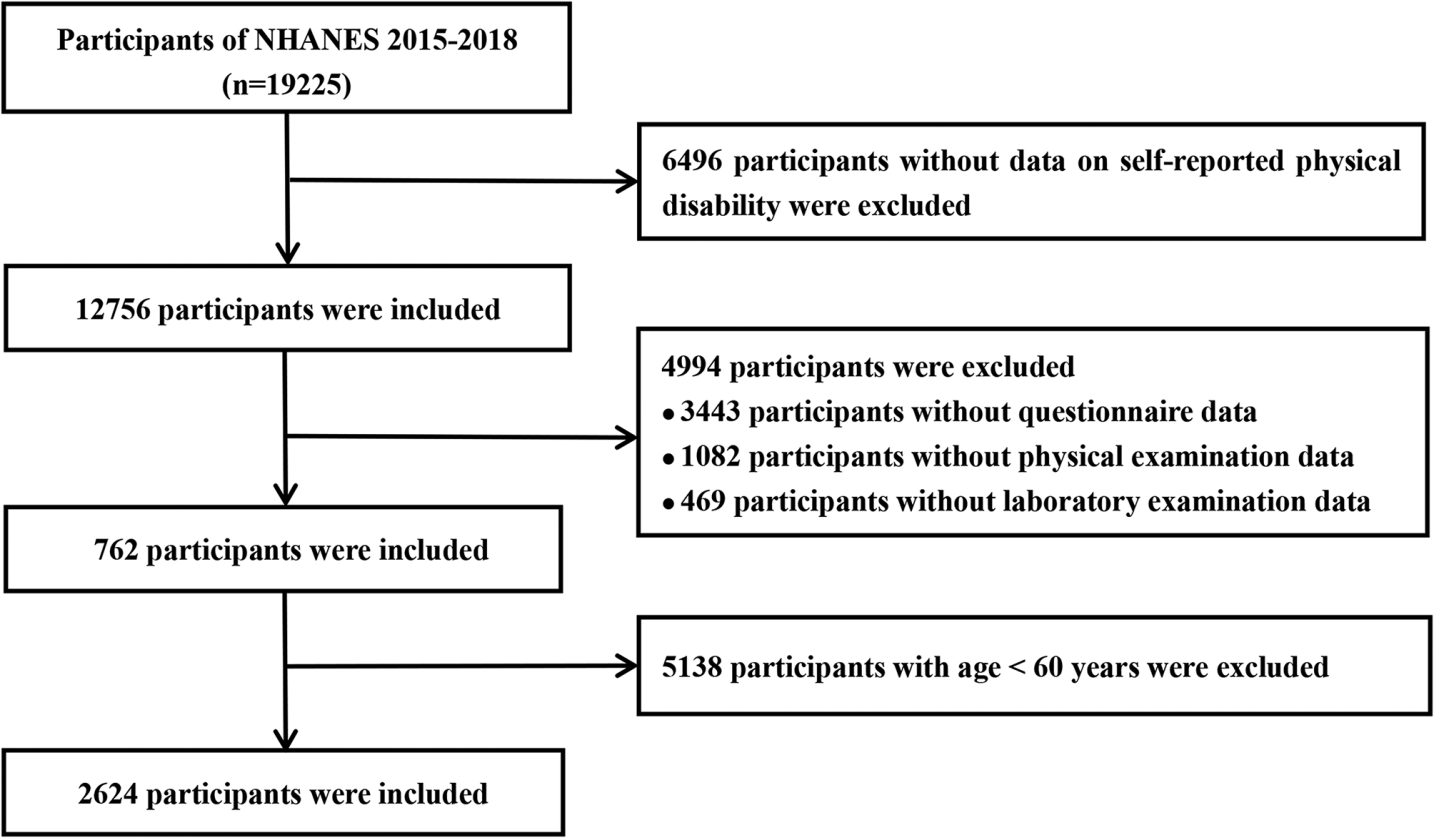
Flowchart showing the selection of study population

The data was derived from NHANES database(2015–2018). The NHANES is a cross-sectional survey conducted by the National Centre for Health Statistics (NCHS). As a large clinical program in the United States, NHANES was designed to investigate the health status of Americans, involving demographic, dietary, health-related questionnaires, physical examination and laboratory data. The NHANES program was approved by the Ethics Review Board of the NCHS. Among 19,225 participants in NHANES 2015–2018, 11,526 participants were excluded due to missing data. Meanwhile, we also excluded 5138 participants aged < 60 years. 2624 participants aged ≥ 60 years were eventually included in our study (Fig. 1). Complete details about NHANES can be accessed from https://www.cdc.gov/nchs/nhanes/index.htm.

### Physical disability measurement

Physical disability was assessed by six questions: “Have serious difficulty hearing (SDH)?”, “Have serious difficulty seeing (SDS)?”, “Have serious difficulty concentrating (SDC)?”, “Have serious difficulty walking (SDW)?”, “Have difficulty dressing or bathing (DDB)?” and “Have difficulty doing errands alone (DDEA)?”. Responses to these questions were “yes” or “no”. Answer yes to one of the above six questions is identified as physical disability.

### Standing height measurement

Standing height (cm) usually was measured with an altimeter. It requires the subject to stand in front of the height meter, the head upright, the back and heel close to the height meter, and then the surveyor read the value on the instrument to obtain the height of the subject. Standing height was categorized into quartiles (< 157.40, 157.40−164.99, 165.00−172.20, > 172.20), and the lowest quartile was considered as the reference group.

### Covariates

Sociodemographic variables included gender (female, male), age (years) and race (Mexican American, Other Hispanic, Non-Hispanic White, Non-Hispanic Black, Other Race). As a continuous variable, age was also categorized into four following groups: 60–64, 65–69, 70–74 and 75 years or older. Health-related variables included diabetes (yes, no), hypertension (yes, no), high cholesterol level (yes, no), trouble sleeping (yes, no), failing kidneys (yes, no), vigorous work activity (yes, no), hepatitis B (yes, no), hepatitis C (yes, no) and feeling depressed (not at all, several days, more than half the days, nearly every day). These variables were obtained by self-report. Physical examination variables included weight (kg), waist circumference (cm), upper leg length (cm), upper arm length (cm), arm circumference (cm), systolic blood pressure (SBP, mmHg) and diastolic blood pressure (DBP, mmHg). Leg and arm measurements were performed on the right side of the body. Measurement would be taken on the left side if a participant had an amputation or other adverse condition. Laboratory examination variables included albumin (mg/L), creatinine (µmol/L), high density lipoprotein cholesterol (HDL-C, mmol/L), total cholesterol (mmol/L), glycohemoglobin (%) and hypersensitive C-reactive protein (Hs-CRP, mg/L). These laboratory indicators were obtained by measuring the serum samples.

### Statistical analyses

We used frequencies, percentages, means and standard deviation to describe variable characteristics. Continuous variables and dichotomous variables were analysed by analysis of variance (ANOVA) and Chi-Square (*χ*^2^) test to calculate differences between four standing height groups. We created three logistic regression models to determine associations between standing height and physical disability among U.S. adults aged 60 years and older. Model 1 was unadjusted; Model 2 Adjusted for gender, age and race based on Model 1; Model 3 additionally adjusted for diabetes, hypertension, high cholesterol level, trouble sleeping, failing kidneys, vigorous work activity, hepatitis B, hepatitis C, feeling depressed, waist circumference, upper leg length, upper arm length, arm circumference, systolic blood pressure, diastolic blood pressure, albumin, creatinine, high density lipoprotein cholesterol, total cholesterol, glycohemoglobin and hypersensitive C-reactive protein based on model 2. Because weight existed severe collinearity with other variables, we did not include weight as an adjusted variable in model 3. The association between standing height and physical disability was further explored by subgroup analyses stratified by gender, age and race. Moreover, we used restricted cubic spline (RCS) model to detect the possible nonlinear dose-response relationship between standing height and physical disability. The significant level is *P* < 0.05. R version 4.1.0 and SPSS version 25.0 were used to perform all statistical analyses.

## Results

### Characteristics of participants

Table 1 showed the characteristics of participants. A total of 2624 participants aged 60 years and older were included in our study, including 1279 (48.7%) females and 1345 (51.3%) males. The mean age of participants was 69.41 ± 6.82 years and 1099 (41.9%) participants reported that they were Non-Hispanic White. For the diseased condition, 722 (27.5%) participants had diabetes mellitus, 1563 (59.6%) participants had hypertension, 1485 (56.6%) participants had high cholesterol level, 169 (6.4%) participants had failing kidneys, 42 (1.6%) participants had hepatitis B, and 52 (2.0%) participants had hepatitis C. Trouble sleeping was found in 861 (32.8%) participants, and 464 (17.7%) participants had vigorous work activity. Among all participants, 1105 (42.1%) participants reported physical disability, of which 459 (17.5%) participants reported SDH, 239 (9.1%) participants reported SDS, 295 (11.2%) participants reported SDC, 622 (23.7%) participants reported SDW, 174 (6.6%) participants reported DDB and 252 (9.6%) participants reported DDEA.

**Table 1:** Characteristics of study population according to levels of standing height (*N* = 2624)

Characteristics	Quartiles of Standing Height (cm)					
	Q1 (< 157.40)	Q2 (157.40−164.99)	Q3 (165.00−172.20)	Q4 (> 172.20)	Total	*P* Value
**Continuous variables[mean ± SD]**						
Age(years)	70.23 ± 6.84	69.06 ± 6.78	69.58 ± 6.96	68.77 ± 6.60	69.41 ± 6.82	0.001
Systolic blood pressure(mmHg)	138.07 ± 20.88	135.63 ± 20.04	135.17 ± 19.03	131.82 ± 17.68	135.18 ± 19.57	< 0.001
Diastolic blood pressure(mmHg)	66.33 ± 15.51	67.56 ± 15.23	68.75 ± 14.00	70.89 ± 13.79	68.37 ± 14.74	< 0.001
Weight(kg)	68.61 ± 15.04	76.44 ± 16.29	81.80 ± 15.84	93.74 ± 18.82	80.10 ± 18.90	< 0.001
Waist circumference(cm)	98.49 ± 13.42	101.46 ± 14.25	103.74 ± 13.23	108.66 ± 14.96	103.07 ± 14.46	< 0.001
Upper leg length(cm)	33.97 ± 2.49	36.34 ± 2.68	38.78 ± 2.46	41.40 ± 2.90	37.61 ± 3.82	< 0.001
Upper arm length(cm)	34.46 ± 1.86	36.57 ± 1.77	38.10 ± 1.63	40.49 ± 1.94	37.39 ± 2.84	< 0.001
Arm circumference(cm)	31.07 ± 4.53	32.35 ± 4.72	32.44 ± 4.10	34.23 ± 4.34	32.52 ± 4.57	< 0.001
Albumin(mg/L)	50.18 ± 318.61	67.59 ± 322.30	77.26 ± 355.60	56.48 ± 221.98	62.85 ± 308.73	0.398
Creatinine(umol/L)	7716.92 ± 5705.46	9116.30 ± 5875.96	10962.96 ± 6389.31	12267.81 ± 6678.79	10005.54 ± 6408.59	< 0.001
HDL-C (mmol/L)	1.54 ± 0.45	1.50 ± 0.46	1.34 ± 0.42	1.30 ± 0.42	1.42 ± 0.45	< 0.001
Total cholesterol(mmol/L)	5.11 ± 1.11	5.06 ± 1.14	4.72 ± 1.11	4.64 ± 1.10	4.88 ± 1.13	< 0.001
Glycohemoglobin (%)	6.19 ± 1.26	6.21 ± 1.29	6.11 ± 1.05	6.15 ± 1.18	6.17 ± 1.20	0.476
HS-CRP (mg/L)	3.88 ± 6.24	4.32 ± 8.29	4.08 ± 7.30	3.72 ± 6.25	4.00 ± 7.07	0.448
**Categorical variables[n(%)]**						
Gender						< 0.001
Female	626(94.8)	469(71.0)	159(24.4)	25(3.8)	1279(48.7)	
Male	34(5.2)	192(29.0)	492(75.6)	627(96.2)	1345(51.3)	
Age group						0.020
60–64 years	180(27.3)	224(33.9)	208(32.0)	219(33.6)	831(37.7)	
65–69 years	151(22.9)	152(23.0)	136(20.9)	159(24.4)	598(22.8)	
70–74 years	130(19.7)	104(15.7)	111(17.0)	125(19.2)	470(17.9)	
≥ 75 years	199(30.1)	181(27.4)	196(30.1)	149(22.8)	725(27.6)	
Race						< 0.001
Mexican American	135(20.5)	103(15.6)	103(15.8)	34(5.2)	375(14.3)	
Other Hispanic	124(18.8)	83(12.6)	69(10.6)	45(6.9)	321(12.2)	
Non-Hispanic White	212(32.1)	258(39.0)	276(42.4)	353(54.1)	1099(41.9)	
Non-Hispanic Black	78(11.8)	135(20.4)	141(21.7)	178(27.3)	532(20.3)	
Other Race	111(16.8)	82(12.4)	62(9.5)	42(6.5)	297(11.3)	
Diabetes						0.016
No	508(77.0)	477(72.2)	465(71.4)	452(69.3)	1902(72.5)	
Yes	152(23.0)	184(27.8)	186(28.6)	200(30.7)	722(27.5)	
Hypertension						0.079
No	243(36.8)	261(39.5)	276(42.4)	281(43.1)	1061(40.4)	
Yes	417(63.2)	400(60.5)	375(57.6)	371(56.9)	1563(59.6)	
High cholesterol level						0.051
No	267(40.5)	272(41.1)	305(46.9)	295(45.2)	1139(43.4)	
Yes	393(59.5)	389(58.9)	346(53.1)	357(54.8)	1485(56.6)	
Trouble sleeping						0.253
No	458(69.4)	426(64.4)	444(68.2)	435(66.7)	1763(67.2)	
Yes	202(30.6)	235(35.6)	207(31.8)	217(33.3)	861(32.8)	
Failing kidneys						0.318
No	622(94.2)	617(93.3)	615(94.5)	601(92.2)	2455(93.6)	
Yes	38(5.8)	44(6.7)	36(5.5)	51(7.8)	169(6.4)	
Vigorous work activity						< 0.001
No	592(89.7)	566(85.6)	513(78.8)	489(75.0)	2160(82.3)	
Yes	68(10.3)	95(14.4)	138(21.2)	163(25.0)	464(17.7)	
Hepatitis B						0.035
No	656(99.4)	646(97.7)	643(98.8)	637(97.7)	2582(98.4)	
Yes	4(0.6)	15(2.3)	8(1.2)	15(2.3)	42(1.6)	
Hepatitis C						0.090
No	653(98.9)	650(98.3)	636(97.7)	633(97.1)	2572(98.0)	
Yes	7(1.1)	11(1.7)	15(2.3)	19(2.9)	52(2.0)	
Feeling depressed						0.017
Not at all	487(73.8)	505(76.4)	522(80.2)	533(81.7)	2047(78.0)	
Several days	109(16.5)	112(17.0)	81(12.5)	78(12.0)	380(14.5)	
More than half the days	35(5.3)	20(3.0)	27(4.1)	23(3.5)	105(4.0)	
Nearly every day	29(4.4)	24(3.6)	21(3.2)	18(2.8)	92(3.5)	
**Self-report physical disability[n(%)]**						
All physical disability(APD)						0.415
No	364(55.2)	391(59.2)	379(58.2)	385(59.0)	1519(57.9)	
Yes	296(44.8)	270(40.8)	272(41.8)	267(41.0)	1105(42.1)	
Serious difficulty hearing(SDH)						< 0.001
No	567(85.9)	569(86.1)	523(80.3)	506(77.6)	2165(82.5)	
Yes	93(14.1)	92(13.9)	128(19.7)	146(22.4)	459(17.5)	
Serious difficulty seeing(SDS)						0.726
No	598(90.6)	595(90.0)	594(91.2)	598(91.7)	2385(90.9)	
Yes	62(9.4)	66(10.0)	57(8.8)	54(8.3)	239(9.1)	
Serious difficulty concentrating(SDC)						0.108
No	576(87.3)	589(89.1)	570(87.6)	594(91.1)	2329(88.8)	
Yes	84(12.7)	72(10.9)	81(12.4)	58(8.9)	295(11.2)	
Serious difficulty walking(SDW)						0.002
No	477(72.3)	490(74.1)	517(79.4)	518(79.4)	2002(76.3)	
Yes	183(27.7)	171(25.9)	134(20.6)	134(20.6)	622(23.7)	
Difficulty dressing or bathing(DDB)						0.266
No	606(91.8)	624(94.4)	611(93.9)	609(93.4)	2450(93.4)	
Yes	54(8.2)	37(5.6)	40(6.1)	43(6.6)	174(6.6)	
Difficulty doing errands alone(DDEA)						< 0.001
No	563(85.3)	598(90.5)	603(92.6)	608(93.3)	2372(90.4)	
Yes	97(14.7)	63(9.5)	48(7.4)	44(6.7)	252(9.6)	

*Abbreviations* HS-CRP: Hypersensitive C-reactive protein; HDL-C: High density lipoprotein cholesterol

Characteristics of all participants were stratified by quartiles of standing height, from which statistically significant differences were found in gender, age, race, systolic blood pressure, diastolic blood pressure, weight, waist circumference, upper leg length, upper arm length, arm circumference, creatinine, HDL-C, total cholesterol, diabetes, vigorous work activity, hepatitis B, feeling depressed, SDH, SDW and DDEA (all *P* < 0.05). Participants with SDW and DDEA had a lower standing height. There were no significant difference in albumin, glycohemoglobin, hypersensitive C-reactive protein, hypertension, high cholesterol level, trouble sleeping, failing kidneys, hepatitis C, SDS, SDC and DDB among quartiles of standing height (all *P* > 0.05).

### Association between standing height and physical disability

**Fig. 2:**
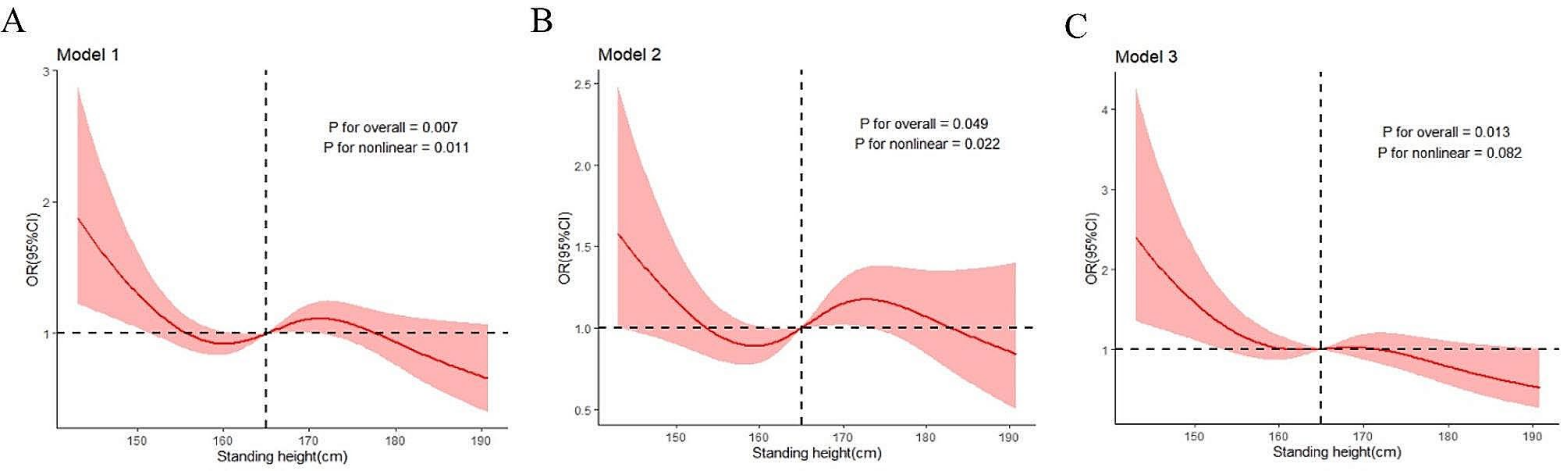
Restricted cubic spline of association between standing height and all physical disability(APD); Model 1: unadjusted; Model 2: Adjusted for gender, age and race; Model 3: adjusted for gender, age, race, diabetes, hypertension, high cholesterol level, trouble sleeping, failing kidneys, vigorous work activity, hepatitis B, hepatitis C, feeling depressed, waist circumference, upper leg length, upper arm length, arm circumference, systolic blood pressure, diastolic blood pressure, albumin, creatinine, high density lipoprotein cholesterol, total cholesterol, glycohemoglobin and hypersensitive C-reactive protein

**Fig. 3:**
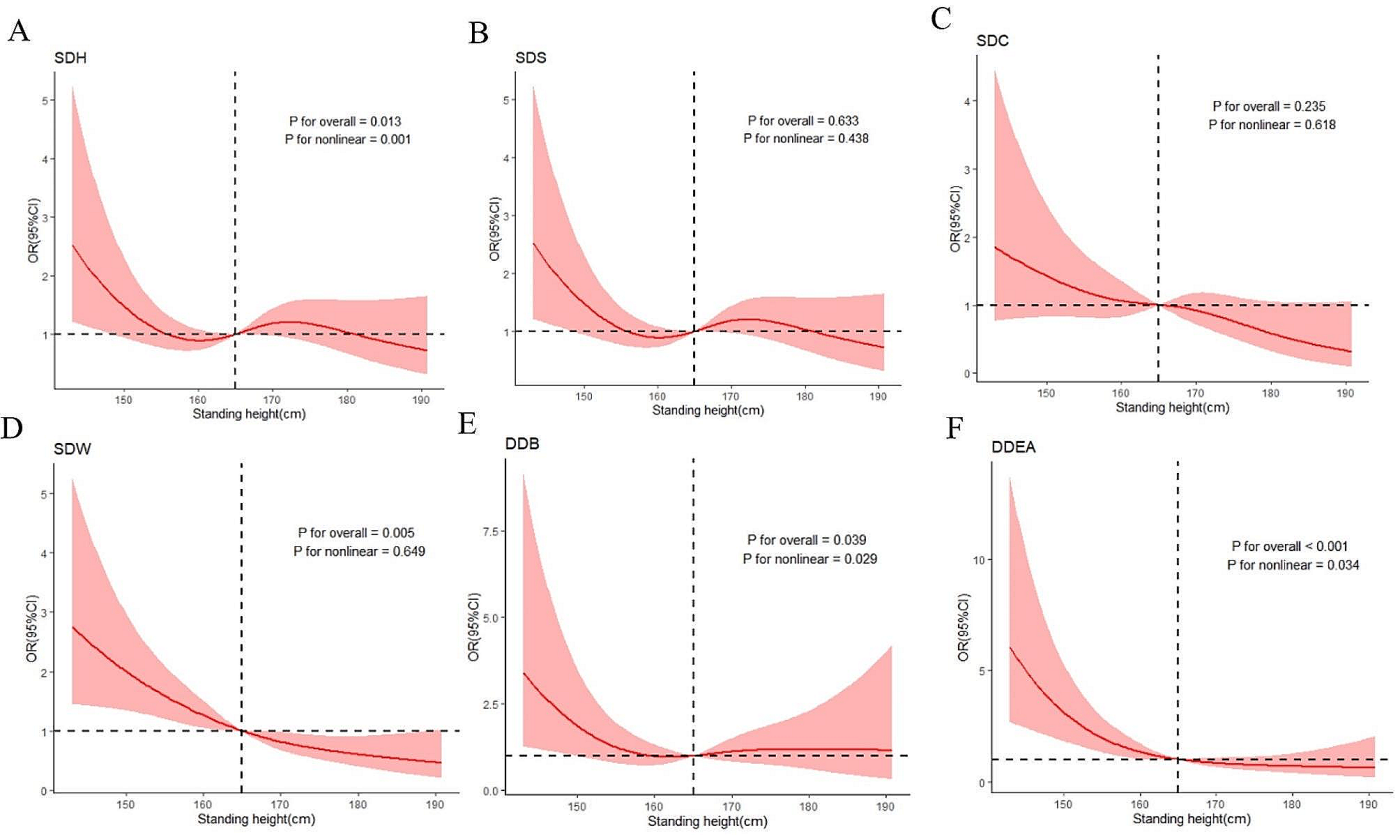
Restricted cubic spline of association between standing height and different types of physical disability after adjusting all confounders; SDH: Serious difficulty hearing; SDS: Serious difficulty seeing; SDC: Serious difficulty concentrating; SDW: Serious difficulty walking; DDB: Difficulty dressing or bathing; DDEA: Difficulty doing errands alone

Standing height was included as a continuous variable for the subsequent analysis. We found standing height was a protective factor for all physical disability (APD) after performing a logistic regression analysis (Table 2). The association was statistically significant in the full-adjusted model (Model 3) (*OR*= 0.976, 95*%CI*: 0.957–0.995). The RCS model results showed a negative linear relationship between standing height and the risk of APD (*P* for nonlinear = 0.082) (Fig. 2). For per 1-cm increase in standing height, the risk of APD decreased by 2.4%. Among the six types of physical disability, standing height was a protective factor for SDW (*OR*= 0.961, 95*%CI*:0.939–0.983) and DDEA (*OR*= 0.944, 95*%CI*:0.915–0.975). These relationships were significant in Model 3. The RCS model results (Fig. 3) also demonstrated a negative linear relationship between standing height and the risk of SDW (*P* for nonlinear = 0.649) and a negative nonlinear relationship between standing height and the risk of DDEA (*P* for nonlinear = 0.034). For every 1-cm increase in standing height, the risk of SDW and DDEA decreased by 3.9% and 5.6%, respectively. There was no significant association between standing height and SDH, SDS, SDC, and DDB after adjusting for all potential confounders.

**Table 2:** Association between standing height and physical disability: models with standing height as a continuous variable

Physical disability	Model 1			Model 2			Model 3	
	OR(95%CI)	*P* Value		OR(95%CI)	*P* Value		OR(95%CI)	*P* Value
APD	0.993(0.985–1.001)	0.074		0.997(0.986–1.009)	0.667		0.976(0.957–0.995)	0.015
SDH	1.020(1.009–1.030)	< 0.001		1.004(0.989–1.020)	0.576		0.988(0.964–1.013)	0.344
SDS	0.993(0.979–1.006)	0.280		0.994(0.975–1.014)	0.572		0.996(0.964–1.028)	0.785
SDC	0.987(0.975–0.999)	0.040		0.980(0.963–0.998)	0.031		0.971(0.941–1.002)	0.066
SDW	0.983(0.974–0.992)	< 0.001		1.004(0.991–1.018)	0.558		0.961(0.939–0.983)	0.001
DDB	0.989(0.974–1.004)	0.161		0.994(0.972–1.017)	0.625		0.981(0.945–1.019)	0.321
DDEA	0.963(0.950–0.976)	< 0.001		0.968(0.949–0.988)	0.001		0.944(0.915–0.975)	< 0.001

*Abbreviations* APD: All physical disability; SDH: Serious difficulty hearing; SDS: Serious difficulty seeing; SDC: Serious difficulty concentrating; SDW: Serious difficulty walking; DDB: Difficulty dressing or bathing; DDEA: Difficulty doing errands alone; Model 1: unadjusted; Model 2: Adjusted for gender, age and race; Model 3: adjusted for gender, age, race, diabetes, hypertension, high cholesterol level, trouble sleeping, failing kidneys, vigorous work activity, hepatitis B, hepatitis C, feeling depressed, waist circumference, upper leg length, upper arm length, arm circumference, systolic blood pressure, diastolic blood pressure, albumin, creatinine, high density lipoprotein cholesterol, total cholesterol, glycohemoglobin and hypersensitive C-reactive protein

### Subgroup analyses

Subgroup analyses of gender, age and race were conducted to further identify the relationship between standing height and physical disability. Subgroup analyses results were showed in Table 3. After adjusting for all potential confounders, the inverse association between standing height and APD was statistically significant in female (*OR* = 0.968, 95*%CI*:0.941–0.997), age ≥ 75 years (*OR* = 0.950, 95*%CI*:0.916–0.984) and the race of Non-Hispanic White (*OR* = 0.967, 95*%CI*:0.939–0.995). In these populations, we also found standing height was a protective factor for SDW and DDEA in Model 3. Moreover, through subgroup analyses, we observed that the negative relationship between standing height and SDC was statistically significant in the age group of 70–74 years (*OR* = 0.909, 95*%CI*:0.839–0.984) and the race of Other Hispanic (*OR* = 0.889, 95*%CI*:0.810–0.975) in model 3.

**Table 3:** Subgroup analyses of association between standing height and physical disability

Physical disability	Model 1			Model 2			Model 3	
	OR(95%CI)	*P* Value		OR(95%CI)	*P* Value		OR(95%CI)	*P* Value
Female								
APD	0.977(0.961–0.994)	0.007		0.990(0.973–1.007)	0.256		0.968(0.941–0.997)	0.030
SDH	0.968(0.943–0.993)	0.012		0.987(0.960–1.014)	0.333		0.976(0.937–1.017)	0.248
SDS	0.999(0.971–1.028)	0.936		1.008(0.978–1.038)	0.619		1.035(0.983–1.089)	0.194
SDC	0.979(0.954–1.005)	0.119		0.983(0.957–1.010)	0.219		0.968(0.924–1.014)	0.167
SDW	0.992(0.974–1.010)	0.374		0.999(0.981–1.019)	0.953		0.962(0.932–0.993)	0.015
DDB	0.966(0.935–0.998)	0.039		0.975(0.942–1.009)	0.148		0.966(0.915–1.020)	0.211
DDEA	0.940(0.915–0.966)	< 0.001		0.950(0.924–0.976)	< 0.001		0.919(0.880–0.959)	< 0.001
Male								
APD	0.995(0.981–1.010)	0.522		1.004(0.988–1.019)	0.645		0.986(0.959–1.012)	0.288
SDH	0.999(0.982–1.017)	0.950		1.013(0.994–1.032)	0.168		0.995(0.964–1.026)	0.739
SDS	0.978(0.953–1.003)	0.090		0.984(0.958–1.010)	0.234		0.975(0.928–1.024)	0.310
SDC	0.971(0.949–0.994)	0.014		0.978(0.954–1.002)	0.068		0.976(0.934–1.020)	0.275
SDW	1.006(0.988–1.024)	0.537		1.008(0.989–1.027)	0.398		0.959(0.927–0.992)	0.016
DDB	1.009(0.979–1.039)	0.580		1.010(0.979–1.042)	0.536		0.989(0.939–1.043)	0.691
DDEA	0.985(0.958–1.013)	0.288		0.989(0.961–1.017)	0.431		0.977(0.930–1.026)	0.342
60–64 years								
APD	1.006(0.992–1.021)	0.405		1.004(0.984–1.024)	0.724		0.988(0.952–1.026)	0.526
SDH	1.035(1.013–1.057)	0.001		1.017(0.986–1.049)	0.286		0.977(0.927–1.030)	0.385
SDS	0.999(0.975–1.024)	0.963		1.001(0.966–1.037)	0.943		1.012(0.953–1.074)	0.705
SDC	0.990(0.968–1.012)	0.361		0.994(0.963–1.026)	0.719		0.999(0.937–1.064)	0.975
SDW	0.998(0.981–1.014)	0.784		1.010(0.987–1.034)	0.385		0.967(0.925–1.011)	0.139
DDB	1.006(0.980–1.034)	0.641		1.012(0.974–1.052)	0.537		1.014(0.944–1.088)	0.711
DDEA	0.977(0.951–1.003)	0.082		0.973(0.938–1.010)	0.145		0.941(0.881–1.006)	0.073
65–69 years								
APD	1.006(0.989–1.023)	0.478		1.011(0.987–1.036)	0.362		1.004(0.960–1.049)	0.870
SDH	1.042(1.016–1.068)	0.001		1.015(0.979–1.052)	0.417		1.024(0.959–1.092)	0.480
SDS	0.998(0.968–1.028)	0.883		1.006(0.963–1.052)	0.777		0.991(0.913–1.076)	0.829
SDC	1.002(0.975–1.029)	0.898		0.997(0.959–1.036)	0.872		1.000(0.926–1.081)	0.991
SDW	0.990(0.972–1.009)	0.318		1.022(0.994–1.051)	0.118		0.999(0.949–1.052)	0.974
DDB	0.982(0.948–1.017)	0.303		0.992(0.944–1.043)	0.759		1.031(0.939–1.131)	0.524
DDEA	0.985(0.955–1.016)	0.337		1.004(0.960–1.050)	0.864		1.000(0.924–1.081)	0.994
70−74years								
APD	0.983(0.965–1.001)	0.067		0.997(0.970–1.025)	0.849		0.974(0.927–1.023)	0.291
SDH	1.009(0.985–1.034)	0.455		0.989(0.953–1.026)	0.559		0.976(0.919–1.037)	0.438
SDS	0.986(0.957–1.015)	0.338		0.988(0.945–1.032)	0.584		0.998(0.926–1.076)	0.963
SDC	0.955(0.926–0.984)	0.003		0.949(0.908–0.993)	0.023		0.909(0.839–0.984)	0.018
SDW	0.973(0.951–0.996)	0.021		0.994(0.960–1.028)	0.716		0.962(0.906–1.020)	0.193
DDB	1.009(0.972–1.048)	0.622		1.016(0.960–1.074)	0.590		0.950(0.863–1.047)	0.301
DDEA	0.968(0.936−1.000)	0.052		0.991(0.943–1.041)	0.719		0.948(0.868–1.035)	0.234
≥ 75 years								
APD	0.980(0.966–0.996)	0.011		0.974(0.952–0.996)	0.022		0.950(0.916–0.984)	0.005
SDH	1.017(1.000−1.034)	0.050		0.994(0.970–1.018)	0.617		0.981(0.944–1.019)	0.323
SDS	0.988(0.964–1.014)	0.361		0.983(0.948–1.021)	0.380		0.981(0.925–1.040)	0.517
SDC	0.996(0.973–1.018)	0.699		0.967(0.935–1.001)	0.054		0.972(0.921–1.025)	0.295
SDW	0.970(0.954–0.987)	0.001		0.986(0.961–1.011)	0.265		0.930(0.893–0.968)	< 0.001
DDB	0.963(0.935–0.992)	0.012		0.961(0.921–1.003)	0.066		0.953(0.892–1.019)	0.953
DDEA	0.944(0.922–0.966)	< 0.001		0.935(0.905–0.967)	< 0.001		0.922(0.875–0.972)	0.003
Mexican American								
APD	1.010(0.986–1.035)	0.420		1.029(0.993–1.067)	0.115		1.038(0.973–1.107)	0.260
SDH	1.045(1.014–1.077)	0.004		1.039(0.994–1.086)	0.092		1.004(0.934–1.080)	0.904
SDS	1.004(0.968–1.041)	0.833		0.989(0.938–1.042)	0.670		1.043(0.953–1.142)	0.358
SDC	0.993(0.954–1.033)	0.720		0.992(0.936–1.051)	0.779		1.042(0.934–1.163)	0.461
SDW	0.983(0.955–1.011)	0.228		1.039(0.995–1.085)	0.083		1.015(0.942–1.094)	0.693
DDB	0.999(0.957–1.043)	0.980		1.032(0.968–1.099)	0.334		1.008(0.911–1.114)	0.880
DDEA	0.965(0.930–1.001)	0.059		0.979(0.927–1.034)	0.454		0.993(0.906–1.088)	0.878
Other Hispanic								
APD	0.980(0.956–1.003)	0.093		0.996(0.958–1.035)	0.830		0.990(0.926–1.059)	0.770
SDH	0.999(0.966–1.033)	0.961		0.987(0.934–1.042)	0.624		0.955(0.879–1.038)	0.276
SDS	0.985(0.950–1.022)	0.431		0.985(0.929–1.044)	0.600		1.040(0.947–1.143)	0.407
SDC	0.979(0.949–1.010)	0.179		0.948(0.902–0.997)	0.037		0.889(0.810–0.975)	0.013
SDW	0.979(0.954–1.005)	0.121		1.021(0.979–1.066)	0.331		0.994(0.917–1.078)	0.891
DDB	0.936(0.894–0.980)	0.005		0.950(0.886–1.018)	0.147		0.880(0.766–1.010)	0.070
DDEA	0.946(0.909–0.986)	0.008		1.000(0.938–1.067)	0.993		0.966(0.863–1.081)	0.547
Non-Hispanic White								
APD	0.990(0.978–1.002)	0.104		0.981(0.963-1.000)	0.052		0.967(0.939–0.995)	0.024
SDH	1.020(1.006–1.035)	0.007		0.991(0.968–1.014)	0.425		0.986(0.952–1.021)	0.422
SDS	0.979(0.958–1.002)	0.069		0.971(0.938–1.005)	0.095		0.945(0.898–0.995)	0.030
SDC	0.988(0.969–1.007)	0.218		0.976(0.947–1.005)	0.103		0.980(0.935–1.026)	0.387
SDW	0.976(0.962–0.990)	0.001		0.984(0.962–1.006)	0.153		0.954(0.921–0.988)	0.009
DDB	0.996(0.971–1.022)	0.744		0.980(0.942–1.019)	0.307		0.973(0.914–1.035)	0.378
DDEA	0.950(0.929–0.973)	< 0.001		0.940(0.907–0.973)	0.001		0.910(0.861–0.962)	0.001
Non-Hispanic Black								
APD	0.985(0.967–1.003)	0.108		0.989(0.963–1.015)	0.405		0.957(0.915-1.000)	0.052
SDH	1.011(0.981–1.042)	0.460		1.010(0.967–1.056)	0.646		0.955(0.887–1.028)	0.224
SDS	1.007(0.975–1.039)	0.674		1.036(0.990–1.085)	0.130		0.997(0.926–1.074)	0.947
SDC	0.979(0.950–1.009)	0.173		0.962(0.922–1.004)	0.073		0.975(0.902–1.053)	0.514
SDW	0.979(0.958–0.999)	0.044		0.985(0.956–1.015)	0.321		0.937(0.891–0.986)	0.012
DDB	1.006(0.970–1.044)	0.753		1.011(0.959–1.066)	0.681		1.017(0.935–1.108)	0.689
DDEA	0.987(0.957–1.018)	0.411		0.994(0.951–1.039)	0.791		0.961(0.894–1.032)	0.270
Other Race								
APD	1.012(0.987–1.038)	0.359		1.024(0.986–1.063)	0.214		0.949(0.886–1.016)	0.133
SDH	1.034(0.998–1.071)	0.068		1.021(0.969–1.077)	0.434		1.002(0.912–1.102)	0.961
SDS	1.055(1.006–1.106)	0.028		1.062(0.993–1.136)	0.079		1.056(0.930–1.200)	0.398
SDC	1.015(0.966–1.067)	0.555		1.032(0.961–1.109)	0.386		0.926(0.785–1.093)	0.365
SDW	1.012(0.981–1.044)	0.437		1.066(1.017–1.117)	0.007		0.943(0.857–1.037)	0.228
DDB	1.023(0.969–1.080)	0.420		1.034(0.957–1.118)	0.396		0.959(0.818–1.126)	0.611
DDEA	0.984(0.943–1.026)	0.452		0.997(0.938–1.059)	0.919		0.929(0.836–1.031)	0.167

*Abbreviations* APD: All physical disability; SDH: Serious difficulty hearing; SDS: Serious difficulty seeing; SDC: Serious difficulty concentrating; SDW: Serious difficulty walking; DDB: Difficulty dressing or bathing; DDEA: Difficulty doing errands alone; Model 1: unadjusted; Model 2: Adjusted for gender, age and race; Model 3: adjusted for gender, age, race, diabetes, hypertension, high cholesterol level, trouble sleeping, failing kidneys, vigorous work activity, hepatitis B, hepatitis C, feeling depressed, waist circumference, upper leg length, upper arm length, arm circumference, systolic blood pressure, diastolic blood pressure, albumin, creatinine, high density lipoprotein cholesterol, total cholesterol, glycohemoglobin and hypersensitive C-reactive protein

## Discussion

Disability is an important cause of affecting the quality of life and causing death among the elderly. As the proportion of the elderly population increases globally, the prevalence of physical disability will increase substantially. In the nationally representative survey, we demonstrated that standing height was a protective factor for APD after adjusting for all potential confounders. For per 1-cm increase in standing height, the risk of APD decreased by 2.4%. In addition, a negative dose-response relationship between standing height and risk of APD was also showed by RCS model. Besides, among six types of physical disability, we also found standing height was a protective factor for SDW and DDEA in the full-adjusted model. Our study provides further support for the relationship between standing height and physical disability among adults aged 60 years and older.

Our findings were in line with previous similar studies reporting the protective effect of standing height on physical disability. A cohort study including 421 rheumatoid arthritis patients from Northern European origin showed that adult height is inversely linked with impairment of joint function and overall disability [16]. In addition, a previous study in Japanese participants found that taller (≥ 170 cm for men and ≥ 160 cm for women) people were 16% less likely to report functional limitation in comparison with shorter (< 155 cm for men and < 145 cm for women) individuals [17]. Moreover, it is widely accepted that fracture is one of the important reasons for physical disability in the elderly. A global longitudinal study of osteoporosis in 52,939 postmenopausal women also demonstrated a negative association between height and upper arm/shoulder and clavicle fractures [18]. For per 10-cm height increment, the risk of upper arm/shoulder and clavicle fractures decreased by 15% and 27%, respectively. In short, the above studies indicated that standing height may be inversely associated with the risk of physical disability in the elderly. The possible biological mechanisms are as follows: Taller people usually have wider bones and may counteract the adverse effects of cortical structural changes, and shorter people with narrower bones may thus be more likely to have fractures and eventually cause physical disability [18, 19].

Our findings are not consistent with several previous studies. A recent cross-sectional investigation among 155 Japanese older adults found that participants with higher height were more likely to report a decline in activities of daily living (ADL) [20]. Including seven prospective studies, a meta-analysis from US, Norway and other European countries in 2016 demonstrated positive association between height and risk of hip fracture [21]. Meanwhile, a cohort study involving 796,081 postmenopausal women with a follow-up period of 8 years also suggested taller women were at increased risk of fracture [22]. These seem to contradict the conclusions of our study. There are several possible reasons for these inconsistent conclusions. First, participants in our study were older than 60 years, while participants in almost all other studies were a mix of younger (under 60 years) and older adults. Second, the role of confounders may contribute to inconsistent results. Previous studies did not adjust some possible confounders, such as SBP, DBP, HDL-C, total cholesterol, glycohemoglobin, etc [23, 24]. Instead, our study considered more potential confounders (24 factors) than others. Thus our findings are even more convincing. Third, different study populations may be one of the reasons why the conclusions are inconsistent. Currently, few studies investigate the relationship between standing height and physical disability in the elderly. More studies are needed to further clarify this relationship in the future.

Our subgroup analyses showed inverse significant relationship between standing height and APD in females in Model 3, but this relationship was not observed in males. Related studies to explain the sex differences are rare. For females, higher height may reduce the risk of APD. More studies should be conducted to explore the cause of sex differences in the future. For age and race, after adjusting for all confounders, the negative association between standing height and APD was statistically significant in participants aged ≥ 75 years and the race of Non-Hispanic White, while other subgroups had no significant association. These associations may not reach statistical significance due to reduced statistical power. More evidence should be explored in a larger sample population in future studies.

Our findings have clinically important implications. With aging populations, the increase in physical disability in older adults is a public health problem worldwide. As this trend develops, more older people will eventually lose their ability to care for themselves. Besides, the increase in physical disability may also lead to an increased risk of many chronic diseases [25]. As an individual basic characteristic, standing height should be considered as a predictor of physical disability in adults aged 60 years and older.

Our study has several strengths. On the one hand, our study adjusted more possible confounders than others. Therefore, our results were more credible. On the other hand, we classified all physical disability (APD) into six categories (SDH, SDS, SDC, SDW, DDB, DDEA) and explored the relationship between standing height and six types of physical disability separately. Thus our study was more comprehensive. Meanwhile, there are some limitations in our study. First, NHANES was a cross-sectional study, so we were difficult to know the causal relationship between standing height and physical disability. In the future, interventional or prospective studies with larger sample sizes should be conducted to further confirm our conclusions. Second, data on physical disability were obtained by self-reporting from participants, which inevitably had a certain degree of subjectivity. Third, more than half participants were excluded from our study due to missing information, which might cause bias.

## Conclusion

Our study suggests that standing height is a protective factor for APD after adjusting for all potential confounders. In addition, among six types of physical disability, inverse association between standing height and SDW and DDEA is also statistically significant in the full-adjusted model. It is necessary to pay close attention to changes in standing height and intervene in time. Future prospective studies with large sample sizes are still needed to verify our findings.

## Data Availability

The datasets used and/or analysed during the current study are available from the corresponding author on reasonable request.

## References

[1] Swai EA, Msuya SE, Moshi H (2023). Children and adolescents with physical disabilities: describing characteristics and disability-related needs in the Kilimanjaro region, north-eastern Tanzania-a cross-sectional survey. BMJ Open.

[2] Deierlein AL, Litvak J, Stein CR (2023). Dietary Quality and Diet-related factors among female adults of Reproductive Age with and without disabilities participating in the National Health and Nutrition Examination Surveys, 2013–2018. J Acad Nutr Diet.

[3] Fong JH (2019). Disability incidence and functional decline among older adults with major chronic diseases. BMC Geriatr.

[4] Yin Z, Shi X, Kraus VB (2014). Gender-dependent association of body mass index and waist circumference with disability in the Chinese oldest old. Obes (Silver Spring).

[5] Okoro CA, Hollis ND, Cyrus AC (2018). Prevalence of disabilities and Health Care Access by disability status and type among adults - United States, 2016. MMWR Morb Mortal Wkly Rep.

[6] Zheng PP, Guo ZL, Du XJ (2022). Prevalence of disability among the Chinese older Population: a systematic review and Meta-analysis. Int J Environ Res Public Health.

[7] World Health Organization World Report on Disability. 2011. [(accessed on 6 December 2021)]. Available online: www.who.int/disabilities/world_report/2011/en/.

[8] Luna-Orozco K, Fernández-Niño JA, Astudillo-García CI (2020). Association between physical disability and incidence of depressive symptoms in older Mexican adults. Biomedica.

[9] Inchai P, Tsai WC, Chiu LT (2021). Incidence, risk, and associated risk factors of stroke among people with different disability types and severities: a national population-based cohort study in Taiwan. Disabil Health J.

[10] Li H, Wang Y, Xue Y (2023). Association of demographics, cardiovascular indicators and disability characteristics with 7-year coronary heart disease incident in persons with disabilities. BMC Public Health.

[11] Gregg Bardenheier BH, Gregg EW, Zhuo X, Cheng YJ, Geiss LS (2014). Association of functional decline with subsequent diabetes incidence in U.S. adults aged 51 years and older: the Health and Retirement Study 1998–2010. Diabetes Care.

[12] García-Peña C, Pérez-Zepeda MU (2017). Validity of knee-estimated height to assess standing height in older adults: a secondary longitudinal analysis of the Mexican Health and Aging Study. J Nutr Health Aging.

[13] Chu AHY, Yuan WL, Loy SL (2021). Maternal height, gestational diabetes mellitus and pregnancy complications. Diabetes Res Clin Pract.

[14] Aune D, Vieira AR, Chan DS (2012). Height and pancreatic cancer risk: a systematic review and meta-analysis of cohort studies. Cancer Causes Control.

[15] Sohail H, Hassan SM, Yaqoob U (2021). The height as an independent risk factor of atrial fibrillation: a review. Indian Heart J.

[16] Chen Y, Yu Z, Packham JC (2013). Influence of adult height on rheumatoid arthritis: association with disease activity, impairment of joint function and overall disability. PLoS ONE.

[17] Fujiwara T, Kondo K, Shirai K (2014). Associations of childhood socioeconomic status and adulthood height with functional limitations among Japanese older people: results from the JAGES 2010 Project. J Gerontol Biol Sci Med Sci.

[18] Compston JE, Flahive J, Hosmer DW (2014). Relationship of weight, height, and body mass index with fracture risk at different sites in postmenopausal women: the global longitudinal study of osteoporosis in women (GLOW). J Bone Min Res.

[19] Moayyeri A, Luben RN, Bingham SA (2008). Measured height loss predicts fractures in middle-aged and older men and women: the EPIC-Norfolk prospective population study. J Bone Min Res.

[20] Tanaka Y, Ando T, Tsuchiya K et al. Height and weight, not body Mass Index, are closely Associated with activities of Daily living in Japanese older adults. Asia Pac J Public Health 2024 Apr 20:10105395241247336. doi: 10.1177/10105395241247336. Epub ahead of print. 10.1177/10105395241247336 38641963

[21] Xiao Z, Ren D, Feng W (2016). Height and risk of hip fracture: a Meta-analysis of prospective cohort studies. Biomed Res Int.

[22] Armstrong ME, Kirichek O, Cairns BJ (2016). Relationship of height to site-specific fracture risk in Postmenopausal Women. J Bone Min Res.

[23] Cochran JM, Siebert VR, Bates J (2021). The relationship between adult height and blood pressure. Cardiology.

[24] Song Y, Liu J, Zhao K (2021). Cholesterol-induced toxicity: an integrated view of the role of cholesterol in multiple diseases. Cell Metab.

[25] Nam S, Kuo YF, Markides KS (2012). Waist circumference (WC), body mass index (BMI), and disability among older adults in latin American and the Caribbean (LAC). Arch Gerontol Geriatr.

